# Pattern of early human-to-human transmission of Wuhan 2019 novel coronavirus (2019-nCoV), December 2019 to January 2020

**DOI:** 10.2807/1560-7917.ES.2020.25.4.2000058

**Published:** 2020-01-30

**Authors:** Julien Riou, Christian L. Althaus

**Affiliations:** 1 Institute of Social and Preventive Medicine, University of Bern, Bern, Switzerland

**Keywords:** 2019-nCoV, emerging infectious disease, mathematical modelling, Wuhan, coronavirus

## Abstract

Since December 2019, China has been experiencing a large outbreak of a novel coronavirus (2019-nCoV) which can cause respiratory disease and severe pneumonia. We estimated the basic reproduction number *R_0_* of 2019-nCoV to be around 2.2 (90% high density interval: 1.4–3.8), indicating the potential for sustained human-to-human transmission. Transmission characteristics appear to be of similar magnitude to severe acute respiratory syndrome-related coronavirus (SARS-CoV) and pandemic influenza, indicating a risk of global spread.

On 31 December 2019, the World Health Organization (WHO) was alerted about a cluster of pneumonia of unknown aetiology in the city of Wuhan, China [1,2]. Only a few days later, Chinese authorities identified and characterised a novel coronavirus (2019-nCoV) as the causative agent of the outbreak [3]. The outbreak appears to have started from a single or multiple zoonotic transmission events at a wet market in Wuhan where game animals and meat were sold [4] and has resulted in 5,997 confirmed cases in China and 68 confirmed cases in several other countries by 29 January 2020 [5]. Based on the number of exported cases identified in other countries, the actual size of the epidemic in Wuhan has been estimated to be much larger [6]. At this early stage of the outbreak, it is important to gain understanding of the transmission pattern and the potential for sustained human-to-human transmission of 2019-nCoV. Information on the transmission characteristics will help coordinate current screening and containment strategies, support decision making on whether the outbreak constitutes a public health emergency of international concern (PHEIC), and is key for anticipating the risk of pandemic spread of 2019-nCoV. In order to better understand the early transmission pattern of 2019-nCoV, we performed stochastic simulations of early outbreak trajectories that are consistent with the epidemiological findings to date.

## Epidemic parameters

Two key properties will determine further spread of 2019-nCoV. Firstly, the basic reproduction number *R_0_* describes the average number of secondary cases generated by an infectious index case in a fully susceptible population, as was the case during the early phase of the outbreak. If *R_0_* is above the critical threshold of 1, continuous human-to-human transmission with sustained transmission chains will occur. Secondly, the individual variation in the number of secondary cases provides further information about the expected outbreak dynamics and the potential for superspreading events [7-9]. If the dispersion of the number of secondary cases is high, a small number of cases may be responsible for a disproportionate number of secondary cases, while a large number of cases will not transmit the pathogen at all. While superspreading always remain a rare event, it can result in a large and explosive transmission event and have a lot of impact on the course of an epidemic. Conversely, low dispersion would lead to a steadier growth of the epidemic, with more homogeneity in the number of secondary cases per index case. This has important implications for control efforts.

## Simulating early outbreak trajectories

In a first step, we initialised simulations with one index case. For each primary case, we generated secondary cases according to a negative-binomial offspring distribution with mean *R_0_* and dispersion *k* [7,8]. The dispersion parameter *k* quantifies the variability in the number of secondary cases, and can be interpreted as a measure of the impact of superspreading events (the lower the value of *k*, the higher the impact of superspreading). The generation time interval *D* was assumed to be gamma-distributed with a shape parameter of 2, and a mean that varied between 7 and 14 days. We explored a wide range of parameter combinations (Table) and ran 1,000 stochastic simulations for each individual combination. This corresponds to a total of 3.52 million one-index-case simulations that were run on UBELIX (http://www.id.unibe.ch/hpc), the high performance computing cluster at the University of Bern, Switzerland.

**Table:** Parameter ranges for stochastic simulations of outbreak trajectories, 2019 novel coronavirus outbreak, China, 2019–2020

Parameter	Description	Range	Number of values explored within the range
*R_0_*	Basic reproduction number	0.8–5.0	22 (equidistant)
*k*	Dispersion parameter	0.0110	20 (equidistant on log_10_ scale)
*D*	Generation time interval (days)	9–11,13,16–19	8 (equidistant)
*n*	Initial number of index cases	1–50	6 (equidistant)
*T*	Date of zoonotic transmission	20 Nov–4 Dec 2019	Randomised for each index case

In a second step, we accounted for the uncertainty regarding the number of index cases *n* and the date *T* of the initial zoonotic animal-to-human transmissions at the wet market in Wuhan. An epidemic with several index cases can be considered as the aggregation of several independent epidemics with one index case each. We sampled (with replacement) *n* of the one-index-case epidemics, sampled a date of onset for each index case and aggregated the epidemic curves together. The sampling of the date of onset was done uniformly from a 2-week interval around 27 November 2019, in coherence with early phylogenetic analyses of 11 2019-nCoV genomes [10]. This step was repeated 100 times for each combination of *R_0_* (22 points), *k* (20 points), *D* (8 points) and *n* (6 points) for a total of 2,112,000 full epidemics simulated that included the uncertainty on *D*, *n* and *T*. Finally, we calculated the proportion of stochastic simulations that reached a total number of infected cases within the interval between 1,000 and 9,700 by 18 January 2020, as estimated by Imai et al. [6]. In a process related to approximate Bayesian computation (ABC), the parameter value combinations that led to simulations within that interval were treated as approximations to the posterior distributions of the parameters with uniform prior distributions. Model simulations and analyses were performed in the R software for statistical computing [11]. Code files are available on https://github.com/jriou/wcov.

## Transmission characteristics of the 2019 novel coronavirus

**Figure 1:**
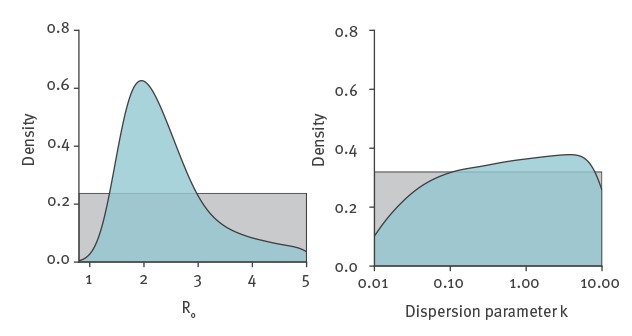
Values of *R_0_* and *k* most compatible with the estimated size of the 2019 novel coronavirus epidemic in China, on 18 January 2020

**Figure 2:**
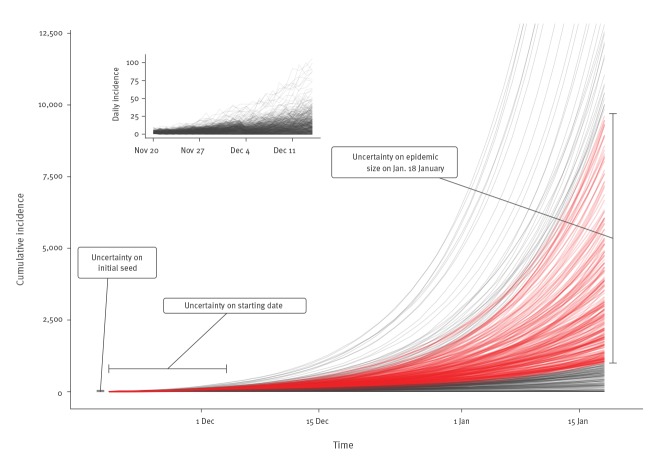
Illustration of the simulation strategy, 2019 novel coronavirus outbreak, China, 2019–2020

In order to reach between 1,000 and 9,700 infected cases by 18 January 2020, the early human-to-human transmission of 2019-nCoV was characterised by values of *R_0_* around 2.2 (median value, with 90% high density interval: 1.4–3.8) (Figure 1). The observed data at this point are compatible with a large range of values for the dispersion parameter *k* (median: 0.54, 90% high density interval: 0.014–6.95). However, our simulations suggest that very low values of *k* are less likely. These estimates incorporate the uncertainty about the total epidemic size on 18 January 2020 and about the date and scale of the initial zoonotic event (Figure 2).

The basic reproduction number *R_0_* quantifies human-to-human transmission. The dispersion parameter *k* quantifies the risk of a superspreading event (lower values of *k* are linked to a higher probability of superspreading). Note that the probability density of *k* implies a log_10_ transformation.

The lines represent the cumulative incidence of 480 simulations with *R_0_ = 1.8* and *k* = 1.13. The other parameters are left to vary according to the Table. Among these simulated epidemics, 54.3% led to a cumulative incidence between 1,000 and 9,700 on 18 January 2020 (in red).

## Comparison with past emergences of respiratory viruses

**Figure 3:**
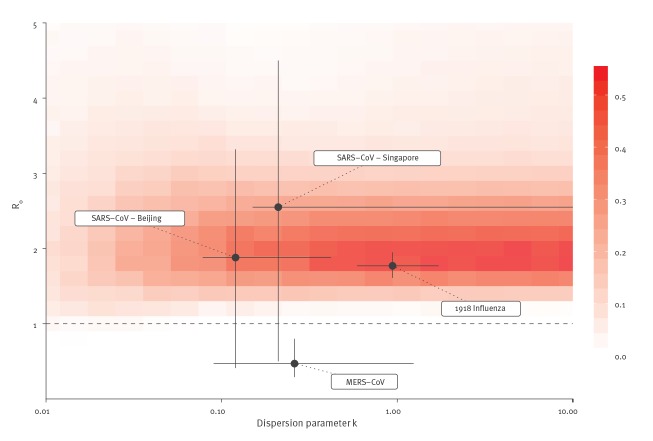
Proportion of simulated epidemics that lead to a cumulative incidence between 1,000 and 9,700 of the 2019 novel coronavirus outbreak, China, on 18 January 2020

MERS: Middle East respiratory syndrome-related coronavirus; SARS: severe acute respiratory syndrome-related coronavirus.

Comparison with other emerging coronaviruses in the past allows to put into perspective the available information regarding the transmission patterns of 2019-nCoV. Figure 3 shows the combinations of *R_0_* and *k* that are most likely at this stage of the epidemic. Our estimates of *R_0_* and *k* are more similar to previous estimates focusing on early human-to-human transmission of SARS-CoV in Beijing and Singapore [7] than of Middle East respiratory syndrome-related coronavirus (MERS-CoV) [9]. The spread of MERS-CoV was characterised by small clusters of transmission following repeated instances of animal-to-human transmission events, mainly driven by the occurrence of superspreading events in hospital settings. MERS-CoV could however not sustain human-to-human transmission beyond a few generations [12]. Conversely, the international spread of SARS-CoV lasted for 9 months and was driven by sustained human-to-human transmission, with occasional superspreading events. It led to more than 8,000 cases around the world and required extensive efforts by public health authorities to be contained [13]. Our assessment of the early transmission of 2019-nCoV suggests that 2019-nCoV might follow a similar path.

This can be interpreted as the combinations of *R_0_* and *k* values most compatible with the estimation of epidemic size before quarantine measures were put in place. As a comparison, we show the estimates of *R_0_* and *k* for the early human-to-human transmission of SARS-CoV in Singapore and Beijing and of 1918 pandemic influenza [7,9,14].

Our estimates for 2019-nCoV are also compatible with those of 1918 pandemic influenza, for which *k* was estimated [14]. Human-to-human transmission of influenza viruses is characterised by *R_0_* values between 1.5 and 2 and a larger value of *k*, implying a more steady transmission without superspreading. The emergence of new strains of influenza, for which human populations carried little to no immunity contrary to seasonal influenza, led to pandemics with different severity such as the ones in1918, 1957 1968 and 2009. It is notable that coronaviruses differ from influenza viruses in many aspects, and evidence for the 2019-nCoV with respect to case fatality rate, transmissibility from asymptomatic individuals and speed of transmission is still limited. Without speculating about possible consequences, the values of *R_0_* and *k* found here during the early stage of 2019-nCoV emergence and the lack of immunity to 2019-nCoV in the human population leave open the possibility for pandemic circulation of this new virus.

## Strengths and limitations

The scarcity of available data, especially on case counts by date of disease onset as well as contact tracing, greatly limits the precision of our estimates and does not yet allow for reliable forecasts of epidemic spread. Case counts provided by local authorities in the early stage of an emerging epidemic are notoriously unreliable as reporting rates are unstable and vary with time. This is due to many factors such as the initial lack of proper diagnosis tools, the focus on the more severe cases or the overcrowding of hospitals. We avoided this surveillance bias by relying on an indirect estimate of epidemic size on 18 January, based on cases identified in foreign countries before quarantine measures were implemented on 23 January. This estimated range of epidemic size relies itself on several assumptions, including that all infected individuals who travelled from Wuhan to other countries have been detected [6]. This caveat may lead to an underestimation of transmissibility, especially considering the recent reports about asymptomatic cases [4]. Conversely, our results do not depend on any assumption about the existence of asymptomatic transmission, and only reflect the possible combinations of transmission events that lead to the situation on 18 January. 

Our analysis, while limited because of the scarcity of data, has two important strengths. Firstly, it is based on the simulation of a wide range of possibilities regarding epidemic parameters and allows for the full propagation on the final estimates of the many remaining uncertainties regarding 2019-nCoV and the situation in Wuhan: on the actual size of the epidemic, on the size of the initial zoonotic event at the wet market, on the date(s) of the initial animal-to-human transmission event(s) and on the generation time interval. As it accounts for all these uncertainties, our analysis provides a summary of the current state of knowledge about the human-to-human transmissibility of 2019-nCoV. Secondly, its focus on the possibility of superspreading events by using negative-binomial offspring distributions appears relevant in the context of emerging coronaviruses [7,8]. While our estimate of *k* remains imprecise, the simulations suggest that very low values of *k* < 0.1 are less likely than higher values < 0.1 that correspond to a more homogeneous transmission pattern. However, values of *k* in the range of 0.1–0.2 are still compatible with a small risk of occurrence of large superspreading events, especially impactful in hospital settings [15,16].

## Conclusions

Our analysis suggests that the early pattern of human-to-human transmission of 2019-nCoV is reminiscent of SARS-CoV emergence in 2002. International collaboration and coordination will be crucial in order to contain the spread of 2019-nCoV. At this stage, particular attention should be given to the prevention of possible rare but explosive superspreading events, while the establishment of sustained transmission chains from single cases cannot be ruled out. The previous experience with SARS-CoV has shown that established practices of infection control, such as early detection and isolation, contact tracing and the use of personal protective equipment, can stop such an epidemic. Given the existing uncertainty around the case fatality rate and transmission, our findings confirm the importance of screening, surveillance and control efforts, particularly at airports and other transportation hubs, in order to prevent further international spread of 2019-nCoV.
