# Author’s correction for Euro Surveill. 2020;25(4)

**DOI:** 10.2807/1560-7917.ES.2020.25.7.20200220c

**Published:** 2020-02-20

**Authors:** Eurosurveillance editorial team

**Affiliations:** 1 European Centre for Disease Prevention and Control (ECDC), Stockholm, Sweden

On request of the authors, the ranges for the generation time and the dispersion parameter in the Table were corrected in the article ‘Pattern of early human-to-human transmission of Wuhan 2019 novel coronavirus (2019-nCoV), December 2019 to January 2020’ by Riou and Althaus, published on 30 January 2020. This correction was made on 17 February 2020.

